# ECOTEC_Online student risk dataset: An open institutional dataset for reproducible student risk analytics in online higher education

**DOI:** 10.1016/j.dib.2026.113289

**Published:** 2026-09-25

**Authors:** Jesus Rafael Hechavarria-Hernandez, Johanna Andrea Navarro-Espinosa, Glenda Blanc-Pihuave, Erika Ascencio-Jordán

**Affiliations:** Universidad Ecotec, Research Department, Samborondón, Guayas, EC092302, Ecuador

**Keywords:** Learning analytics, Educational data mining, Student risk analytics, Institutional analytics, Reproducible research, Responsible AI, Class imbalance, FAIR data

## Abstract

The ECOTEC_Online Student Risk Dataset contains anonymized institutional records from online higher education programs at Universidad ECOTEC, Ecuador. The dataset comprises 12,632 student-level records, with exactly one record per unique student, and 18 public variables covering demographic, academic, admission, financial, and institutional information relevant to student risk and institutional analytics in digital learning environments. Universidad ECOTEC provided the research team with an institutionally anonymized dataset prepared in accordance with its approved institutional protocol. The public repository is organized around the finalized anonymized dataset and includes variable-level metadata, data-validation and descriptive-analysis scripts, reproducible Logistic Regression and Random Forest benchmark scripts and outputs, supplementary visualizations, software-environment specifications, and FAIR-oriented repository documentation. TARGET_RISK is a binary variable derived during study-level data curation from restricted STUDENT_TYPE and GRADUATION_DATE information. Operationally, Class 1 denotes students who were not recorded as Alumni and had no valid GRADUATION_DATE recorded in the institutional snapshot; it should not be interpreted as confirmed educational risk or as a prospectively observed dropout outcome. It represents a contemporaneous administrative proxy based on recorded completion status rather than a prospectively observed dropout or retention outcome. Potential reuse scenarios include learning analytics, educational data mining, institutional analytics, class-imbalanced classification, explainable artificial intelligence, machine-learning benchmarking, educational decision-support research, responsible AI, and exploratory fairness-aware analyses using the demographic and institutional subgroup variables retained after anonymization. The dataset and associated reproducibility resources are publicly archived in Zenodo as version 1.0.4 (DOI: 10.5281/zenodo.22015963) [1].

Table 1 Specifications Table

**Table:** 

Subject	*Computer Sciences*
Specific subject area	Learning analytics, educational data mining, and reproducible AI datasets for student risk analytics
Type of data	Table; CSV data; Text; Figure
Data collection	The dataset was derived from institutional academic management information associated with online higher education programs at Universidad ECOTEC, Ecuador. Universidad ECOTEC provided the research team with an institutionally anonymized dataset prepared in accordance with its approved institutional protocol. The finalized public release contains 12,632 unique student records and 18 variables covering demographic, academic, admission, financial, and institutional information. Public analytical scripts operate on the finalized anonymized dataset to perform structural validation, generate descriptive outputs, and reproduce the reported machine-learning benchmarks; they do not perform or reconstruct the institutional anonymization process.
Data source location	Institution: Universidad ECOTEC City/Town/Region: Samborondón, Guayas Country: Ecuador Latitude and longitude: −2.1445, −79.8654
Data accessibility	Repository name: Zenodo Repository version: v1.0.4 Data identification number (DOI): 10.5281/zenodo.22015963 Direct repository URL: https://doi.org/10.5281/zenodo.22015963 Associated GitHub repository: https://github.com/jrhechavarriah/ECOTEC_Online_Student_Risk_Dataset Instructions for accessing these data: The dataset and all associated reproducibility resources are publicly accessible through the Zenodo repository (DOI: 10.5281/zenodo.22015963). The corresponding GitHub repository contains the same archived release synchronized with Zenodo to ensure version consistency, long-term preservation, and reproducible access to all repository resources, under an open-science framework aligned with the FAIR Guiding Principles [1,2]. Repository version: v1.0.4. Accessed 19 August 2026.
Related research article	None.

## 1 Value of the Data

• These data provide a real-world anonymized institutional dataset from online higher education, integrating demographic, academic, admission, financial, and institutional variables relevant to student academic progression and institutional analytics.
• The repository provides a reproducible research resource comprising the finalized anonymized dataset, data-validation and descriptive-analysis scripts, reproducible benchmark machine-learning scripts and outputs, metadata documentation, software-environment specifications, and FAIR-oriented repository resources that facilitate transparent computational reuse, validation, and methodological reproducibility [[1], [2], [3]].
• The dataset supports reproducible research using real-world institutional data in learning analytics, educational data mining, institutional analytics, class-imbalanced classification, explainable artificial intelligence, machine-learning benchmarking, educational decision-support research, and exploratory fairness-aware analyses based on the demographic and institutional subgroup variables retained after anonymization [4,5].
• Because the repository includes the publicly accessible finalized anonymized dataset, data-validation and descriptive-analysis scripts, reproducible benchmark scripts and outputs, metadata documentation, software-environment specifications, and persistent DOI-based archiving, it provides a reusable reference resource for comparative methodological studies, reproducible educational AI research, and transparent evaluation of machine-learning workflows. Fairness-related analyses should be interpreted within the scope of the anonymized demographic and institutional variables retained in the public release.
• Reuse of TARGET_RISK should preserve its operational administrative definition: Class 1 denotes students who were not recorded as Alumni and had no valid GRADUATION_DATE recorded in the institutional snapshot. This class should not be interpreted as a confirmed educational-risk group or as a prospectively observed dropout outcome.

## 2 Background

The increasing use of digital learning environments in higher education has generated growing interest in institutional datasets that support reproducible research in learning analytics, educational data mining, institutional educational analytics, and responsible artificial intelligence research practices [2,3]. Although several benchmark educational datasets have been released, such as the Open University Learning Analytics Dataset (OULAD), publicly accessible institutional datasets that combine comprehensive metadata, transparent anonymization documentation, executable analytical resources, benchmark outputs, and versioned reproducibility support remain relatively limited [[4], [5], [6]].

The ECOTEC_Online Student Risk Dataset was compiled from institutional academic management systems associated with online higher education programs at Universidad ECOTEC, Ecuador. The repository was organized to provide anonymized demographic, academic, financial, admission, and institutional variables associated with student risk analytics under real-world educational conditions. The repository structure was additionally organized following FAIR-oriented data management practices [2].

Several publicly available educational datasets have substantially advanced research in learning analytics and educational data mining, including the Open University Learning Analytics Dataset (OULAD), which combines demographic information with virtual learning environment interactions from distance education courses [6], and the Student Performance dataset, which integrates demographic, social, and academic variables from secondary education to support predictive modeling [7,8]. Although these resources have become important benchmarks for educational machine-learning research, they differ substantially in educational level, institutional context, geographical setting, variable composition, and reproducibility support. The ECOTEC_Online Student Risk Dataset complements these resources by providing anonymized institutional administrative records from fully online higher education programs in Latin America together with comprehensive metadata documentation, data-validation and descriptive-analysis scripts, reproducible benchmark machine-learning scripts and outputs, synchronized GitHub–Zenodo dissemination, software-environment specifications, and FAIR-oriented repository resources designed to facilitate transparent computational reuse.

The repository includes the finalized anonymized public dataset, metadata documentation, data-validation and descriptive-analysis scripts, reproducible benchmark machine-learning scripts and outputs, supplementary visualizations, software-environment specifications, and FAIR-oriented repository documentation intended to facilitate reproducibility and reuse in educational AI research environments.

## 3 Data Description

The complete FAIR-oriented dataset repository is publicly archived in Zenodo [1]. The repository contains the finalized anonymized public dataset, variable-level metadata, data-validation and descriptive-analysis scripts, reproducible benchmark scripts and outputs, supplementary visualizations, software-environment specifications, and FAIR-oriented repository documentation supporting transparent reuse and computational reproducibility in online higher education analytics. The corresponding GitHub v1.0.4 release is archived through Zenodo, providing persistent DOI-based identification and long-term preservation of the version used in this study [1,2]. The finalized public dataset contains no machine-readable null or blank cells across the 12,632 records and 18 released variables. This statement refers specifically to the completeness of the finalized public CSV and should not be interpreted as evidence that every corresponding upstream institutional attribute was necessarily observed before institutional preparation.

### 3.1 FAIR-oriented data management and reproducibility

The repository has been organized to support the FAIR (Findable, Accessible, Interoperable, and Reusable) data principles through explicit repository resources and metadata [2,3]. Findability is supported through persistent DOI assignment by Zenodo, standardized citation metadata (CITATION.cff), and a version-controlled GitHub repository synchronized with the corresponding Zenodo release. Accessibility is provided through public access to the Zenodo archive and associated GitHub repository. Interoperability is supported at the syntactic and documentation levels through the use of a widely supported CSV representation together with structured variable-level metadata and an accompanying codebook that define the released schema, variable meanings, coding conventions, and units of measurement. Reusability is facilitated through the CC BY 4.0 license, variable-level metadata and provenance documentation, comprehensive repository documentation (README.md and DATASET_CARD.md), data-validation and descriptive-analysis scripts, reproducible benchmark scripts and outputs, computational-environment specifications, and publicly archived versioned releases that support transparent computational reuse and long-term preservation. Beyond these FAIR-oriented provisions, computational integrity and reproducibility are further supported through a release-specific SHA256SUMS.txt manifest containing SHA-256 checksums for the archived dataset and repository resources. The benchmark software environment is documented through requirements-lock.txt, which records the principal package versions used in the final benchmark validation environment, together with environment_versions.txt, which records the associated Python and platform information.

### 3.2 Dataset overview

Table 2 summarizes the principal characteristics of the publicly released dataset. The dataset comprises 12,632 anonymized records corresponding to 12,632 unique students and includes 18 public variables, including the binary target variable TARGET_RISK. Each student appears exactly once in the extracted cohort. The dataset therefore represents a cross-sectional student-level institutional snapshot rather than a longitudinal dataset containing repeated observations across degree programs or enrollment periods.

**Table 2:** General characteristics of the ECOTEC_Online Student Risk Dataset. Summary statistics describing the size, structure, target variable, observation unit, and completeness of the publicly released dataset.

Item	Value
Records	12,632
Variables	18
Observation Unit	One unique student (one record per student)
Binary target variable	TARGET_RISK
Classes	0 / 1
Missingness	0.00%

### 3.3 Target variable and observation unit

The observation unit is one unique student, represented by exactly one record in the restricted institutional extraction and in the anonymized public dataset.

TARGET_RISK is a binary variable derived during study-level data curation rather than a native field contained in the restricted 42-variable institutional extraction. Its construction used two restricted administrative source variables, STUDENT_TYPE and GRADUATION_DATE, according to the following deterministic rule: TARGET_RISK = 0 when the student was classified as STUDENT_TYPE = ``Alumni'' or when a valid GRADUATION_DATE was recorded; TARGET_RISK = 1 when the student was not classified as Alumni and no valid graduation date was recorded. For target derivation, a GRADUATION_DATE was considered valid when the restricted institutional field contained a non-empty value that could be parsed as a calendar date; no additional institutional consistency or academic-performance criteria were applied to determine date validity.

No grade threshold, academic-performance cutoff, weighted score, predictive model, or multivariable institutional risk index contributed to target construction. Application of this operational rule assigned 691 records (5.47%) to TARGET_RISK = 0 and 11,941 records (94.53%) to TARGET_RISK = 1.

Both STUDENT_TYPE and GRADUATION_DATE were excluded from the public release after target derivation and were not used as benchmark predictors. TARGET_RISK should therefore be interpreted as a contemporaneous administrative proxy based on the completion-related administrative status recorded in the institutional snapshot as of April 10, 2026, rather than as a prospectively observed dropout or retention outcome. In particular, TARGET_RISK = 1 does not imply that a student subsequently dropped out; it indicates only that neither Alumni status nor a valid graduation date was recorded under the target-construction rule as of the April 10, 2026 institutional snapshot (Table 3).

**Table 3:** Distribution of TARGET_RISK classes. Frequency and percentage distribution of the binary administrative target included in the public dataset.

Class	Operational meaning	Records	Percentage (%)
0	Alumni status and/or valid graduation date recorded	691	5.47
1	Not recorded as Alumni and no valid graduation date recorded	11,941	94.53
Total	—	12,632	100.00

**Note**: TARGET_RISK is a contemporaneous administrative proxy derived from restricted student-status and graduation information. Class labels should not be interpreted as prospectively observed dropout or retention outcomes.

To provide readers with a concise overview of the variables included in the publicly released dataset without requiring consultation of the accompanying metadata files, Table 4 summarizes the principal variable categories, the number of variables within each category, representative examples, and the corresponding missingness in the finalized public dataset. Complete variable definitions, coding schemes, and metadata are provided in the accompanying *variable_dictionary.xlsx* and *codebook.pdf* files distributed with the repository.

**Table 4:** Summary of variable categories included in the publicly released ECOTEC_Online Student Risk Dataset. Complete variable definitions, coding schemes, and metadata are provided in *variable_dictionary.xlsx* and *codebook.pdf* distributed with the repository.

Variable category	No. of variables	Representative variables	Missingness (%)
Record identifier	1	PUBLIC_RECORD_ID	0.0
Academic	3	FACULTY, DEGREE_PROGRAM, CURRICULUM_YEAR	0.0
Demographic	5	GENDER, AGE, MARITAL_STATUS, ETHNICITY, DISABILITY	0.0
Institutional	3	INSTITUTION_TYPE, NATIONALITY_COUNTRY, REGION	0.0
Admission	2	TRANSFER_STUDENT_FLAG, ONLINE_ADMISSION_TYPE	0.0
Financial	3	PAYMENT_PLAN, PAYMENT_DEFERRAL_TYPE, SCHOLARSHIP_NAME	0.0
Target variable	1	TARGET_RISK	0.0
**Total**	18	—	0.0

**Note:** Missingness percentages refer to machine-readable null or empty cells in the finalized public release. Substantive negative or zero-valued administrative states are not treated as missing data.

### 3.4 Repository organization

The repository is organized around the finalized anonymized public dataset and the resources required for transparent computational reuse. The data/ directory contains the canonical public dataset, ecotec_online_student_risk.csv. Variable-level metadata and documentation are provided in the metadata/ directory through variable_dictionary.xlsx and codebook.pdf.

The scripts/ directory contains the shared validation utilities, descriptive figure-generation script, Logistic Regression and Random Forest benchmark scripts, and the files documenting runtime dependencies and the final benchmark validation environment. Model-specific machine-readable outputs are stored in the results/ directory, while the figures/ directory contains the descriptive visualizations generated from the finalized public dataset.

Repository-level documentation, integrity-verification resources, and configuration are provided through README.md, DATASET_CARD.md, CITATION.cff, SHA256SUMS.txt, LICENSE, .gitignore, and .gitattributes. The public repository contains 26 files in total. Table 5 summarizes the archived resources and their analytical roles.

**Table 5:** Repository inventory summarizing repository resources, corresponding input datasets, expected output files, and their roles within the reproducible computational workflow.

Folder/File	Input	Output	Description
data/ecotec_online_student_risk.csv	—	Canonical public dataset	Finalized anonymized institutional dataset used as the canonical input for all released public analytical workflows.
metadata/variable_dictionary.xlsx	Dataset variables	Metadata documentation	Variable definitions, data types, categorical encodings, and missing-value representations.
metadata/codebook.pdf	Dataset variables	Metadata documentation	Dataset structure and variable-category documentation.
scripts/generate_dataset_figures.py	data/ecotec_online_student_risk.csv	figures/class_distribution.png; figures/feature_categories.png; figures/subgroup_distribution.png	Descriptive figure-generation script.
scripts/baseline_random_forest.py	data/ecotec_online_student_risk.csv	results/random_forest/metrics.csv; classification_report.txt; confusion_matrix.csv	Random Forest computational reproducibility baseline.
scripts/baseline_logistic_regression.py	data/ecotec_online_student_risk.csv	results/logistic_regression/metrics.csv; classification_report.txt; confusion_matrix.csv	Logistic Regression computational reproducibility baseline.
scripts/utils.py	Shared by validation, descriptive-analysis, and benchmark scripts	Utility functions	Shared utility functions supporting dataset validation, feature preparation, metric calculation, and reproducible analytical workflows.
scripts/requirements.txt	—	Runtime dependency specification	Specifies the principal compatible Python runtime dependencies required by the released validation, descriptive-analysis, and benchmark scripts.
scripts/requirements-lock.txt	Final benchmark validation environment	Recorded package versions	Records the principal package versions captured in the final benchmark validation environment: NumPy 2.0.2, Pandas 2.2.2, SciPy 1.16.3, Scikit-learn 1.6.1, Joblib 1.5.3, and Matplotlib 3.10.0.
scripts/environment_versions.txt	Final benchmark validation environment	Computational-environment metadata	Documents the associated Python/platform information and principal package versions captured during final benchmark validation.
results/random_forest/metrics.csv	scripts/baseline_random_forest.py	Benchmark results	Random Forest benchmark performance metrics.
results/random_forest/classification_report.txt	scripts/baseline_random_forest.py	Benchmark results	Random Forest classification report.
results/random_forest/confusion_matrix.csv	scripts/baseline_random_forest.py	Benchmark result	Random Forest confusion matrix (rows = actual classes; columns = predicted classes).
results/logistic_regression/metrics.csv	scripts/baseline_logistic_regression.py	Benchmark results	Logistic Regression benchmark performance metrics.
results/logistic_regression/classification_report.txt	scripts/baseline_logistic_regression.py	Benchmark results	Logistic Regression classification report.
results/logistic_regression/confusion_matrix.csv	scripts/baseline_logistic_regression.py	Benchmark result	Logistic Regression confusion matrix (rows = actual classes; columns = predicted classes).
figures/class_distribution.png	scripts/generate_dataset_figures.py	Visualization	TARGET_RISK class distribution visualization.
figures/feature_categories.png	scripts/generate_dataset_figures.py	Visualization	Feature-category distribution visualization.
figures/subgroup_distribution.png	scripts/generate_dataset_figures.py	Visualization	Demographic subgroup distribution visualization.
README.md	Repository	Documentation	Repository overview, installation instructions, and reproducibility documentation.
DATASET_CARD.md	Repository	Documentation	FAIR-oriented dataset documentation and intended reuse scenarios.
CITATION.cff	Repository	Citation metadata	Citation metadata for GitHub and Zenodo integration.
SHA256SUMS.txt	Final archived repository resources	Integrity-verification manifest	Release-specific SHA-256 checksums supporting file-level integrity verification of the archived release.
LICENSE	Repository	License	CC BY 4.0 license governing the public repository materials, including the dataset, documentation, metadata, analytical scripts, benchmark resources, and supplementary outputs.
.gitignore	Repository	Version-control configuration	Git version-control exclusion configuration.
.gitattributes	Repository	Version-control configuration	Defines repository-level Git attributes, including consistent treatment of text-file line endings.

***Note***: For direct computational traceability, scripts/generate_dataset_figures.py generates figures/class_distribution.png, figures/feature_categories.png, and figures/subgroup_distribution.png. The script scripts/baseline_logistic_regression.py generates results/logistic_regression/metrics.csv, classification_report.txt, and confusion_matrix.csv, whereas scripts/baseline_random_forest.py generates the corresponding three files under results/random_forest/. These mappings provide a direct link between each released computational result, its generating script, and its archived output file.

## 4 Experimental Design, Materials and Methods

### 4.1 Institutional context

The dataset was compiled from online higher education programs at Universidad ECOTEC, Ecuador. Institutional academic management systems associated with enrollment, academic performance, student progression, financial records, and virtual learning processes were used as primary data sources. Data extraction procedures were conducted under institutional authorization and privacy-preserving protocols.

### 4.2 Data acquisition

The research dataset originated from institutional academic information systems supporting online higher education programs at Universidad ECOTEC. Upstream extraction and institutional preparation were performed before delivery of the research dataset to the authors; the research team did not access or reconstruct the underlying pre-anonymization database queries. The institutional source integrated demographic, academic, financial, admission, and administrative information relevant to student risk and institutional analytics in online higher education.

Before delivery to the research team, Universidad ECOTEC subjected the institutional data to its approved preparation and anonymization process. The institutional authorization for publication and scientific sharing certifies that the dataset delivered for research corresponds to an anonymized version prepared under the protocol approved for the XAILA-SR project. The research team therefore received an institutionally anonymized research dataset and did not access the pre-anonymization institutional source data. Subsequent study-level curation was conducted on this anonymized research dataset, whereas the public reproducibility workflows operate on the finalized 18-variable public release. Because the institutional preparation process occurred before delivery, the earliest auditable cohort available to the authors begins with the 12,632-record restricted research extract described below.

The institutionally prepared research extract delivered to the research team comprised the complete institutional student population within the defined scope of the study, namely students recorded in Universidad ECOTEC's online higher education programs in the institutional administrative snapshot as of April 10, 2026, totaling 12,632 unique students. No student records belonging to this defined source population were excluded before delivery to the research team. Accordingly, the institutional source-population size and the delivered research-extract size were both 12,632. Although the research team did not access the pre-anonymization source database or reconstruct the underlying extraction queries, this limitation concerns the technical upstream extraction process and does not imply uncertainty regarding the size or completeness of the defined study cohort. The Alumni status and GRADUATION_DATE information used in the study-level derivation of TARGET_RISK refer to the administrative state recorded as of that cutoff date.

As summarized in Table 6, a record-level audit of the restricted institutional extraction confirmed 12,632 rows and 12,632 unique institutional student identifiers. No duplicate student identifiers were identified, and no student was represented more than once across degree programs or enrollment periods. The resulting cohort therefore contains one observation per unique student and should be interpreted as a cross-sectional student-level extraction rather than as a repeated-measures or longitudinal dataset. No record-level exclusions were applied by the research team at this stage.

**Table 6:** Cohort construction and record retention from the complete institutionally defined source cohort delivered to the research team to the final public release.

Stage	Records entering	Records excluded	Records retained	Variables	Description
Institutionally prepared research extract delivered by Universidad ECOTEC	12,632	0	12,632	42	Complete institutional student population within the defined study scope as of April 10, 2026; one record per unique student.
Study-level record audit	12,632	0	12,632	42	Verification identified 12,632 unique student-record identifiers, no duplicated identifiers, no exact duplicate rows, and no repeated student observations.
Target derivation and public variable curation	12,632	0	12,632	18 public variables	TARGET_RISK was derived; restricted source variables were excluded; the institutional identifier was replaced; and selected variables were excluded or generalized for public dissemination.
Final anonymized public release (v1.0.4)	12,632	0	12,632	18	Canonical public dataset archived in GitHub/Zenodo.

**Note**: Universidad ECOTEC performed institutional preparation and anonymization before delivering the research dataset to the authors. The delivered 42-variable research extract comprised the complete institutional student population within the defined study scope as of April 10, 2026 (*n* = 12,632), and no student records within that defined population were excluded before delivery. The research team did not access the pre-anonymization source database or reconstruct the underlying extraction queries; however, this does not affect the documented completeness of the delivered study cohort. “Records excluded” in subsequent rows refers exclusively to study-level record removal after delivery of the research dataset.

As further shown in Table 6, subsequent study-level target derivation, identifier replacement, variable selection, and generalization changed the representation and dimensionality of the data but did not alter the student cohort. Accordingly, all 12,632 records present in the earliest auditable research extract were retained in the final 18-variable public release.

The restricted institutional extraction did not contain a native TARGET_RISK field. The public target was subsequently derived during study-level data curation from STUDENT_TYPE and GRADUATION_DATE before construction of the finalized public release. Both source variables were excluded from the released dataset after target derivation.

Because the research team did not access the pre-anonymization institutional source data, the prevalence and handling of missingness in those upstream records cannot be independently quantified from the research files available to the authors. Within the restricted 42-variable research extract available to the study team, the absence of a recorded GRADUATION_DATE was not treated as unresolved missingness requiring statistical imputation because the presence or absence of a valid graduation date forms part of the operational definition of TARGET_RISK. GRADUATION_DATE itself is not included in the finalized public dataset.

The finalized anonymized public dataset was exported in interoperable tabular format and organized together with variable-level metadata, descriptive-analysis scripts, reproducible benchmark scripts and outputs, software-environment specifications, and FAIR-oriented repository documentation. These resources are publicly archived in the synchronized GitHub–Zenodo [1].

### 4.3 Data integration and preprocessing

The dataset provided to the research team had already undergone institutional preparation and anonymization by Universidad ECOTEC in accordance with its approved institutional protocol. Accordingly, the public analytical workflow begins with the finalized anonymized dataset distributed as *data/ecotec_online_student_risk.csv*; it does not perform institutional anonymization or reconstruct the restricted institutional data-preparation process.

The finalized anonymized dataset constitutes the canonical public input for all released analytical workflows. The public repository does not rely on a separate processed-data copy. The released Python scripts operate directly on this dataset to perform structural validation, generate descriptive outputs, and execute the model-specific transformations required for the reproducible benchmark analyses. The public repository is therefore organized around the finalized anonymized dataset and the analytical workflows that operate directly on it.

Validation of the finalized public dataset confirmed 12,632 records, 18 variables, 12,632 unique PUBLIC_RECORD_ID values, no duplicated rows, and zero null cells. Here, “zero null cells” refers specifically to the absence of machine-readable null or empty values across the 12,632 × 18 cells of the finalized public CSV. This statement describes the completeness of the released CSV only. Because the research team did not access the pre-anonymization source records, no inference is made regarding the historical completeness of individual source attributes before institutional preparation. The finalized public release contains no dedicated categories or sentinel values representing unknown, unavailable, missing, or unspecified information. Substantive administrative values such as SCHOLARSHIP_NAME = “No Scholarship” and PAYMENT_DEFERRAL_TYPE = 0 are valid observed states rather than missing-value codes; for PAYMENT_DEFERRAL_TYPE, 0 explicitly denotes no payment deferral. No records were removed during the reported public analytical workflows because no duplicated rows, missing target values, or missing values in the released public variables were detected.

The released analytical and reproducibility scripts were implemented in Python using Pandas [9], NumPy [10], and Scikit-learn [11], together with the additional runtime dependencies documented in the public repository. *requirements.txt* specifies the principal compatible runtime dependencies, *requirements-lock.txt* records the package versions captured in the final benchmark validation environment, and *environment_versions.txt* documents the associated computational environment.

The benchmark pipelines instantiate defensive missing-value safeguards: a median *SimpleImputer* for numerical predictors and a most-frequent-category *SimpleImputer* for categorical predictors. These components are executed during pipeline fitting and transformation but encounter no missing predictor values in the finalized public dataset; consequently, zero numerical values and zero categorical values were imputed during the reported benchmark executions. The transformations materially applied during benchmark generation were the separation of TARGET_RISK from the predictor matrix, exclusion of PUBLIC_RECORD_ID, one-hot encoding of categorical predictors, numerical standardization for Logistic Regression, and class weighting during model fitting. Numerical scaling was not applied to Random Forest.

Detailed model configurations, train-test partitioning, class-imbalance handling, and performance metrics are described in the Benchmark Reproducibility Resources subsection.

### 4.4 Data anonymization

Universidad ECOTEC provided the research team with an anonymized institutional dataset prepared in accordance with its approved institutional protocol. The institutional authorization for publication and scientific sharing certifies that the version delivered for research and intended for scientific dissemination had undergone institutional anonymization before release to the research team.

The restricted institutional extract used for this study comprised 42 variables containing academic, demographic, financial, admission, and administrative information. The audited extract did not contain student names, national identification numbers, email addresses, or telephone numbers. Variables that were unnecessary for the scientific objectives of the public release or that retained greater institutional or disclosure sensitivity were excluded during public-data preparation.

As part of preparation of the public release, the institutional student-record identifier was replaced through a one-to-one mapping with a release-specific PUBLIC_RECORD_ID. This transformation preserved the one-record-per-student structure of the institutional cohort while removing the original institutional identifier from the public release. The final public dataset contains 12,632 records and 12,632 unique PUBLIC_RECORD_ID values. No public crosswalk linking PUBLIC_RECORD_ID to the original institutional identifier is released. PUBLIC_RECORD_ID is intended only to uniquely reference records within the released dataset and should not be interpreted as a longitudinal person-level linkage key across academic periods, independent institutional extracts, or future dataset releases.

The resulting public release comprises 18 variables selected to retain analytical value for learning analytics, educational data mining, institutional analytics, and student risk analytics while reducing disclosure and re-identification risk [4,5]. Selected quasi-identifying variables were retained only at levels of granularity considered appropriate for scientific dissemination. For the remaining released variables, no additional aggregation or generalization is claimed unless explicitly described below; they are reported in their standardized public categorical or numerical form.

Detailed geographic information was reviewed and harmonized during public-data preparation and subsequently generalized to Ecuador's four natural regions: Coastal Region, Highlands Region, Amazon Region, and Insular Region. Province-, canton-, and sector-level information is not included in the public dataset. Country-specific nationality categories were independently generalized to Ecuadorian and International, reducing disclosure risk associated with sparsely represented nationality categories while retaining the analytically relevant distinction between domestic and international students. AGE was retained as an integer rather than grouped into age bands, and DISABILITY was retained as a binary indicator without higher-granularity disability information. No formal quantitative combination-level disclosure-risk analysis—such as k-anonymity, l-diversity, minimum joint-cell-size analysis, exhaustive quasi-identifier uniqueness screening, or differential privacy—was performed by the research team on the public release. In particular, rare joint combinations involving AGE, DEGREE_PROGRAM, ETHNICITY, DISABILITY, REGION, SCHOLARSHIP_NAME, and other potentially quasi-identifying fields were not exhaustively quantified as a separate formal privacy analysis. Accordingly, the documented anonymization and generalization measures should be interpreted as disclosure-risk-reduction procedures rather than as a formal privacy guarantee or evidence of zero re-identification risk.

The public dataset includes three non-monetary financial and payment-related institutional variables: PAYMENT_PLAN, PAYMENT_DEFERRAL_TYPE, and SCHOLARSHIP_NAME. PAYMENT_PLAN and SCHOLARSHIP_NAME are categorical administrative descriptors, whereas PAYMENT_DEFERRAL_TYPE corresponds to the institutional source field TIPO_DIFIRIMIENTO and represents the recorded payment-deferral duration in months, with 0 indicating no payment deferral. No tuition amounts, financial transaction histories, banking information, debt balances, personal income data, or monetary scholarship amounts are present in the public release. The complete released categories for PAYMENT_PLAN and SCHOLARSHIP_NAME, together with the observed PAYMENT_DEFERRAL_TYPE month values and their frequencies, are documented in the accompanying codebook and machine-readable variable dictionary. These three variables should be interpreted as institutional administrative descriptors rather than as direct measures of individual socioeconomic status.

The restricted variables used to derive TARGET_RISK—STUDENT_TYPE and GRADUATION_DATE—were used for target derivation in this study and were excluded from the public release thereafter. Neither variable is included in the public predictor matrix or available to the benchmark models. The publicly released scripts operate on the finalized anonymized dataset and support data validation, descriptive analysis, and reproducible benchmark analyses.

Public dissemination of the anonymized dataset was formally authorized by Universidad ECOTEC in accordance with its institutional anonymization protocol and the applicable Ecuadorian personal-data protection requirements. The resulting public dataset constitutes the finalized anonymized release used for the descriptive analyses and reproducible benchmark experiments reported in this study.

### 4.5 Variable encoding and formatting

The repository includes tabular datasets in comma-separated values (.csv) format. Variable descriptions, categorical representations, missingness information, and data-level metadata are documented in variable_dictionary.xlsx and the accompanying codebook.pdf. Categorical variables retain standardized institutional representations in the finalized public dataset, while numerical variables are stored in interoperable formats compatible with common statistical and machine-learning environments.

### 4.6 Benchmark reproducibility resources

The repository includes reproducible baseline scripts implementing Logistic Regression and Random Forest classifiers using Scikit-learn [11]. These models are provided as computational reproducibility baselines for the released cross-sectional dataset and are not intended to represent prospectively validated dropout-prediction models or operational early-warning systems.

After excluding PUBLIC_RECORD_ID and TARGET_RISK, the benchmark models used the remaining 16 public variables as predictors. The data were partitioned using a stratified 70% training / 30% testing split, corresponding to 8842 training records and 3790 test records, with random_state = 42. Because each student contributes exactly one record, the same student cannot occur in both partitions. This benchmark evaluation used a single fixed stratified holdout partition. No repeated train–test resampling or cross-validation was performed, and variability across alternative random seeds was not evaluated. Consequently, small numerical differences between the Logistic Regression and Random Forest benchmarks should not be interpreted as evidence of a stable performance advantage of one model over the other across repeated samples or alternative partitions. No direct label-definition leakage was identified in the released benchmark predictor set. However, indirect proxy associations remain possible, and temporal leakage cannot be excluded for prospective use because the predictors and TARGET_RISK originate from the same cross-sectional institutional snapshot as of April 10, 2026. Consequently, the benchmark results should be interpreted as cross-sectional computational reproducibility baselines rather than as evidence of prospective predictive validity.

Prior to model fitting, the benchmark pipelines validated the public input for duplicate records, missing target values, and missing predictor values. No duplicate records or missing target values were detected, no records were removed, and the finalized public predictor matrix contained no missing cells. The pipelines nevertheless include defensive preprocessing components for reproducibility and robust execution. Categorical predictors pass through a most-frequent SimpleImputer followed by one-hot encoding, whereas numerical predictors pass through a median SimpleImputer. Because no predictor values were missing in the released dataset, both imputers replaced zero values during the reported executions.

Numerical predictors were additionally standardized for Logistic Regression, whereas no numerical scaling was applied to Random Forest. Both classifiers used *class_weight = "balanced"* to account for the strong target-class imbalance. Class weighting was applied internally by each Scikit-learn estimator during model fitting on the training subset. No oversampling, undersampling, SMOTE, or other synthetic sample-generation procedure was used. The original observed class distribution was therefore preserved in both the stratified training and test subsets. Logistic Regression was configured as *LogisticRegression (max_iter=1000, random_state=42, class_weight="balanced")*, and Random Forest as *RandomForestClassifier (n_estimators=300, random_state=42, class_weight="balanced", n_jobs=−1)*. No hyperparameter optimization, feature selection, post-hoc threshold optimization, grid search, or randomized search was performed; the reported models therefore represent fixed computational baselines rather than optimized performance estimates. All classifier parameters not explicitly specified in these constructors retained the default values of the archived Scikit-learn version (1.6.1), as documented in requirements-lock.txt.

The two restricted variables used to construct the target, STUDENT_TYPE and GRADUATION_DATE, are not included in the public predictor matrix. Each of the remaining 16 public predictors was reviewed against the target-construction rule and its documented variable definition to determine whether it (i) participated directly in target assignment, (ii) represented a derived form of either target-defining source variable, or (iii) directly encoded the resulting binary label. None of the 16 predictors met these criteria. An additional predictor-level check of the released dataset confirmed that no single released predictor deterministically reproduced TARGET_RISK across all 12,632 observations. Thus, no direct label-definition leakage was identified in the benchmark predictor set. Nevertheless, because the public predictors and the target originate from the same institutional snapshot, indirect or proxy associations with completion status may remain, and temporal leakage cannot be excluded for prospective prediction.

Benchmark performance is reported using accuracy, class-specific precision, recall, and F1-score, together with class support, macro-F1, balanced accuracy, ROC-AUC, and confusion matrices. Table 7 summarizes the principal aggregate benchmark metrics, Table 8 reports class-specific performance, and Table 9 reports the imbalance-aware metrics and confusion-matrix counts. The corresponding machine-readable outputs are archived in the *results/logistic_regression/* and *results/random_forest/* directories.

**Table 7:** Benchmark performance results generated by the publicly released baseline machine-learning workflows. Performance metrics are reported exclusively to demonstrate computational reproducibility and should not be interpreted as validated institutional decision-support models.

Benchmark model	Training	Test	Accuracy	Class-1 F1-score	ROC–AUC
Logistic Regression	8842	3790	0.941	0.968	0.989
Random Forest	8842	3790	0.980	0.989	0.984

**Abbreviations**: ROC–AUC, Area Under the Receiver Operating Characteristic Curve. Performance metrics correspond to the publicly released reproducibility benchmarks generated using a single fixed stratified 70%/30% train–test split with a random seed of 42. Class 1 denotes students who, as of the April 10, 2026 institutional snapshot, were not recorded as Alumni and had no valid GRADUATION_DATE recorded; it should not be interpreted as a prospectively observed dropout outcome. Repeated resampling and cross-validation were not performed. These baseline models are included exclusively as computational reproducibility resources and are not intended for operational educational decision support. Source files: results/logistic_regression/metrics.csv and results/random_forest/metrics.csv, generated by scripts/baseline_logistic_regression.py and scripts/baseline_random_forest.py, respectively.

**Table 8:** Class-specific benchmark performance on the stratified test subset.

Model	Class	Precision	Recall	F1-score	Support
Logistic Regression	0	0.478	0.952	0.637	207
Logistic Regression	1	0.997	0.940	0.968	3583
Random Forest	0	0.832	0.787	0.809	207
Random Forest	1	0.988	0.991	0.989	3583

**Note**: Class-specific metrics were calculated on the same 3790-record stratified test subset used for the aggregate benchmark metrics reported in Table 7. Source files: results/logistic_regression/classification_report.txt and results/random_forest/classification_report.txt, generated by the corresponding benchmark scripts. Because these results derive from a single fixed holdout partition, and Class 0 contains only 207 observations in the test subset, differences between models should be interpreted descriptively and not as evidence of stable comparative superiority across alternative partitions.

**Table 9:** Imbalance-aware metrics and confusion matrices for the reproducibility benchmarks.

Model	Balanced Accuracy	Macro-F1	Actual 0 → Pred. 0	Actual 0 → Pred. 1	Actual 1 → Pred. 0	Actual 1 → Pred. 1
Logistic Regression	0.946	0.802	197	10	215	3368
Random Forest	0.889	0.899	163	44	33	3550

**Note**: Confusion-matrix entries are presented with actual classes as rows and predicted classes as columns. Source files: balanced accuracy and macro-F1 are reported in the model-specific metrics.csv files, while confusion-matrix counts are reported in the corresponding confusion_matrix.csv files under results/logistic_regression/ and results/random_forest/. All files are generated by the corresponding released benchmark scripts.

The class-specific results reveal important differences that are not apparent from overall accuracy or ROC-AUC alone. Logistic Regression achieved high Class-0 recall (0.952) but substantially lower Class-0 precision (0.478), with 215 Class-1 observations incorrectly assigned to Class 0. Random Forest achieved substantially higher Class-0 precision (0.832) and Class-0 F1-score (0.809), although its Class-0 recall was lower (0.787). Both classifiers showed very high performance for the majority Class 1, operationally defined as students who, as of the April 10, 2026 institutional snapshot, were not recorded as Alumni and had no valid graduation date recorded. The contrast among class-specific metrics, balanced accuracy, macro-F1, and conventional aggregate measures illustrates why no single metric should be used to infer practical utility under the strongly imbalanced target distribution.

These benchmarks are intended exclusively as computational reproducibility baselines. They have not undergone prospective or temporal validation, probability-calibration assessment, threshold optimization for institutional objectives, decision-curve or utility analysis, external validation, impact assessment, or deployment testing. Furthermore, TARGET_RISK is a contemporaneous administrative proxy rather than a prospectively observed educational outcome. Accordingly, the reported results should not be interpreted as evidence of prospective early-warning capability, validated institutional predictive performance, institutional readiness, operational decision-support readiness, or suitability for decisions affecting individual students.

The computational environment supporting reproducibility of the public analytical workflows is documented through three complementary files. *requirements.txt* specifies the principal compatible runtime dependencies, *requirements-lock.txt* records the corresponding package versions captured in the final benchmark validation environment, and *environment_versions.txt* documents the associated computational environment. Together with the released validation, descriptive-analysis, and benchmark scripts, these files provide the software information required to reproduce and independently verify the reported public analyses.

For end-to-end public computational reproduction, the finalized anonymized CSV serves directly as the canonical input. The documented workflow sequentially executes scripts/generate_dataset_figures.py, scripts/baseline_logistic_regression.py, and scripts/baseline_random_forest.py. These scripts regenerate the public-data-derived descriptive figures and the model-specific benchmark outputs in the documented figures/ and results/ directories. Documentary tables and the cohort-construction audit are not computational outputs of the public dataset and therefore are not regenerated by this workflow.

## Limitations

The released dataset originates from a single higher education institution in Ecuador and therefore reflects the organizational, academic, administrative, and online-education characteristics of that institutional context. The institutionally prepared research extract delivered by Universidad ECOTEC comprised the complete student population within the defined study scope as of April 10, 2026, totaling 12,632 unique students; no student records belonging to this defined population were excluded before delivery to the research team. Accordingly, uncertainty regarding pre-delivery record selection does not apply to the defined institutional cohort. Nevertheless, cohort completeness within this institutional scope does not imply external representativeness beyond Universidad ECOTEC or beyond its online higher education context. These institutional-context constraints should therefore be considered when interpreting the dataset and when transferring analytical findings or computational workflows to other universities, educational systems, student populations, or academic settings.

A central limitation concerns the interpretation of TARGET_RISK. The target is a contemporaneous administrative proxy derived from Alumni status and recorded graduation information within the same institutional snapshot as the public predictors, corresponding to the administrative state recorded as of April 10, 2026. It is not a prospectively observed dropout or retention outcome and does not provide a defined predictor-before-outcome time horizon. Students assigned to TARGET_RISK = 1 should therefore not be interpreted as confirmed future dropouts; this class indicates only that, as of the April 10, 2026 institutional snapshot, the student was not recorded as Alumni and no valid graduation date was recorded under the target-construction rule. Although the target-defining source variables, STUDENT_TYPE and GRADUATION_DATE, were excluded from the public predictor matrix and no direct label-definition leakage was identified, indirect proxy associations with completion status may remain. The high cross-sectional discrimination observed in the benchmarks may therefore partly reflect contemporaneous predictors that capture or proxy institutional states associated with completion, even though none of the released predictors directly or deterministically reproduces TARGET_RISK. In addition, because the predictors and TARGET_RISK originate from the same cross-sectional institutional snapshot as of April 10, 2026, temporal leakage cannot be excluded for prospective prediction. Consequently, the reported benchmark results should be interpreted only as cross-sectional computational reproducibility baselines and not as evidence of prospective dropout or retention prediction, early-warning capability, causal inference, operational predictive validity, or readiness for institutional decision making.

The dataset represents a cross-sectional student-level institutional snapshot containing one record per unique student and does not include repeated observations of students across academic periods or degree programs. Consequently, it should not be interpreted as a longitudinal panel and cannot be used to examine within-student temporal trajectories. Because the public dataset does not establish a longitudinal predictor-before-outcome sequence, the reported random stratified benchmark split does not constitute temporal validation. CURRICULUM_YEAR represents the curriculum-plan year and should not be interpreted as the student's observation year or academic cohort. Because PUBLIC_RECORD_ID is a release-specific record identifier rather than a documented longitudinal linkage key, its persistence across future independent dataset releases should not be assumed. The public dataset therefore does not support person-level longitudinal linkage across releases or academic periods.

Because no formal quantitative combination-level disclosure-risk analysis was performed on the public release, residual re-identification risk arising from rare combinations of retained quasi-identifying attributes cannot be formally excluded. The documented anonymization and generalization procedures should therefore be interpreted as measures intended to reduce disclosure risk rather than as a formal guarantee of zero re-identification risk.

External transferability may also be affected by inter-institutional differences in admission procedures, academic progression and completion requirements, scholarship and payment policies, student-support services, program structures, data-recording practices, and the design and maturity of online learning environments. Such differences may shift predictor distributions and alter the interpretation of analogous administrative outcomes. Models developed using this dataset should therefore not be transferred directly to other institutions without local variable harmonization, verification of target-definition equivalence, external validation, subgroup assessment, and, where appropriate, recalibration.

Finally, the target distribution is strongly imbalanced, with 691 records (5.47%) assigned to class 0 and 11,941 records (94.53%) assigned to class 1. For this reason, overall accuracy alone is insufficient for evaluating benchmark performance. The repository therefore reports class-specific precision, recall, and F1-score together with macro-F1, balanced accuracy, ROC-AUC, and confusion matrices. These metrics improve the transparency of benchmark evaluation but do not overcome the aforementioned limitations regarding target interpretation, temporal validity, and external generalizability.

## Ethics Statement

The dataset used in this study consists of anonymized institutional records from the online higher education administrative systems of Universidad ECOTEC, Ecuador. Universidad ECOTEC formally certified, through its Institutional Authorization for the Publication and Scientific Sharing of Anonymized Data, dated June 24, 2026, that the dataset delivered to the research team corresponds to an anonymized version prepared in accordance with the institutional protocol approved for the XAILA-SR project and the applicable provisions of the Ecuadorian Organic Law on Personal Data Protection [12].

The institutional authorization further certifies that the version intended for scientific publication contains no direct personal identifiers or information that would reasonably permit individual identification. The research team therefore received an institutionally anonymized research dataset and did not access the pre-anonymization institutional source data.

Universidad ECOTEC formally authorized the scientific use of the anonymized dataset for research, methodological validation, reproducibility, academic dissemination, and related scientific outputs. The authorization also permits publication, preservation, and sharing of the anonymized dataset through recognized scientific repositories and academic data infrastructures, provided that the anonymization and data-protection conditions established in the approved institutional protocol are maintained.

Under Universidad ECOTEC's institutional procedures, formal research ethics committee review was not required for this study because it involved secondary analysis of institutional administrative records that had been anonymized before being provided to the research team. The authors did not recruit participants, interact with students for research purposes, collect new identifiable personal data, or access the pre-anonymization institutional source records. Accordingly, no separate ethics committee approval or waiver was required for the present secondary-data study. The scientific use, publication, preservation, and sharing of the anonymized dataset were formally authorized by Universidad ECOTEC through the Institutional Authorization for the Publication and Scientific Sharing of Anonymized Data dated June 24, 2026. This institutional authorization constitutes the documented institutional basis for use and dissemination of the anonymized data and should not be interpreted as an ethics committee approval or waiver.

The signed institutional authorization has been submitted to the journal as supplementary material for editorial and peer-review verification. It is not included in the open GitHub–Zenodo repository because it contains institutional signatures and administrative information. Access to the authorization may also be requested from the corresponding author, subject to Universidad ECOTEC's authorization and applicable institutional, privacy, and administrative requirements.

The public analytical scripts operate exclusively on the finalized anonymized dataset and do not perform or reconstruct the institutional anonymization process. Public dissemination of the dataset and associated reproducibility resources was conducted in accordance with the institutional authorization, applicable data-protection requirements, and the journal's requirements for ethical and transparent data reporting.

## CRediT Author Statement

**Jesus Rafael Hechavarria-Hernandez:** Conceptualization, Methodology, Software, Validation, Formal analysis, Data curation, Writing – Original draft, Visualization, Supervision; **Johanna Andrea Navarro-Espinosa:** Project administration, Data curation, Investigation, Writing – Review & Editing; **Glenda Blanc-Pihuave:** Investigation, Resources, Data curation, Writing – Review & Editing; **Erika Ascencio-Jordán:** Investigation, Resources, Validation, Writing – Review & Editing.

All authors read and approved the final manuscript.

## Appendix Supplementary materials

Image, application 1
