# Supplementary material for: ECOTEC_Online student risk dataset: An open institutional dataset for reproducible student risk analytics in online higher education
Source: Data Brief. 2026 Sep 25;69:113289. doi: 10.1016/j.dib.2026.113289 (PMC13673050; doi:10.1016/j.dib.2026.113289)
Supplement: Supplementary file 1 [file mmc1.pdf]

Universidad Ecotec

Samborondón, junio 24 de 2026

CARTA DE AUTORIZACIÓN INSTITUCIONAL PARA LA PUBLICACIÓN Y COMPARTICIÓN CIENTÍFICA DE DATOS ANONIMIZADOS

Por medio de la presente, la Universidad Tecnológica ECOTEC certifica que ha autorizado el uso científico del conjunto de datos denominado *ECOTEC_Online_Student_Risk_Dataset* desarrollado en el marco del proyecto de investigación "**Explainable AI-Driven Learning Analytics for Student Retention in Online Higher Education (XAILA-SR)**" con código **2026IAITFIACN_005** con la participación de los investigadores:

1. Johanna Navarro Espinosa
2. Jesús Rafael Hechavarria Hernandez
3. Erika del Pilar Ascencio Jordán
4. Glenda del Rocio Blanc Pihuave

La Universidad ECOTEC deja constancia de que:

1. El conjunto de datos fue obtenido a partir de información institucional utilizada exclusivamente para fines de investigación científica, innovación y desarrollo tecnológico.
2. El conjunto de datos entregado a los investigadores corresponde a una versión anonimizada conforme al protocolo institucional aprobado para el proyecto XAILA-SR y a las disposiciones aplicables de la Ley Orgánica de Protección de Datos Personales del Ecuador.
3. La versión destinada a publicación científica no contiene identificadores personales directos ni información que permita razonablemente la identificación individual de estudiantes, docentes o miembros de la comunidad universitaria.
4. La Universidad ECOTEC autoriza el uso del conjunto de datos anonimizado para la elaboración de artículos científicos, ponencias, tesis, informes técnicos, modelos analíticos, desarrollos tecnológicos y demás productos académicos derivados del proyecto XAILA-SR.
5. La Universidad ECOTEC autoriza la publicación, preservación y compartición del conjunto de datos anonimizado en repositorios científicos, infraestructuras de datos de investigación y plataformas académicas reconocidas nacional e internacionalmente, siempre que se mantengan las condiciones de anonimización y protección establecidas en el protocolo aprobado.
6. La institución respalda la adopción de los principios *FAIR (Findable, Accessible, Interoperable and Reusable)* y las buenas prácticas internacionales de gestión de datos de investigación, promoviendo la transparencia, reproducibilidad y reutilización responsable del conocimiento científico.

En consecuencia, la Universidad Tecnológica ECOTEC autoriza formalmente la difusión científica del conjunto de datos anonimizado "*ECOTEC Online Student Risk Dataset*", así

como su utilización para fines de investigación, validación metodológica, reproducibilidad científica, formación académica y desarrollo de soluciones basadas en evidencia, en concordancia con las políticas institucionales y la normativa vigente.

Para constancia de lo anterior, se suscribe la presente certificación.

Edgar Salas, Ph.D
Director ECOTEC Online
Universidad ECOTEC

Unofficial English Translation for Editorial and Peer-Review Purposes

The following is an English translation of the original institutional authorization issued and signed in Spanish by Universidad ECOTEC on June 24, 2026. The signed Spanish-language document remains the authoritative original. This translation is provided solely to facilitate editorial and peer-review verification and should not be construed as a certified translation.

Samborondón, June 24, 2026

INSTITUTIONAL AUTHORIZATION LETTER FOR THE PUBLICATION AND SCIENTIFIC SHARING OF ANONYMIZED DATA

Through this letter, Universidad Tecnológica ECOTEC certifies that it has authorized the scientific use of the dataset entitled ECOTEC_Online Student Risk Dataset, developed within the framework of the research project "Explainable AI-Driven Learning Analytics for Student Retention in Online Higher Education (XAILA-SR)", project code 2026IAITFIACN_005, with the participation of the following researchers:

1. Johanna Navarro Espinosa
2. Jesús Rafael Hechavarria Hernandez
3. Erika del Pilar Ascencio Jordan
4. Glenda del Rocio Blanc Pihuave

Universidad ECOTEC hereby certifies that:

1. The dataset was obtained from institutional information used exclusively for scientific research, innovation, and technological development purposes.
2. The dataset provided to the researchers corresponds to an anonymized version, prepared in accordance with the institutional protocol approved for the XAILA-SR project and with the applicable provisions of Ecuador's Organic Law on Personal Data Protection.
3. The version intended for scientific publication contains no direct personal identifiers or information that would reasonably permit the individual identification of students, faculty members, or other members of the university community.
4. Universidad ECOTEC authorizes the use of the anonymized dataset for the preparation of scientific articles, conference papers, theses, technical reports, analytical models, technological developments, and other academic outputs derived from the XAILA-SR project.
5. Universidad ECOTEC authorizes the publication, preservation, and sharing of the anonymized dataset through scientific repositories, research data infrastructures, and academic platforms recognized nationally and internationally, provided that the anonymization and data-protection conditions established in the approved institutional protocol are maintained.
6. The institution supports the adoption of the FAIR principles (Findable, Accessible, Interoperable, and Reusable) and internationally recognized good practices for research-data management, thereby promoting transparency, reproducibility, and the responsible reuse of scientific knowledge.

Accordingly, Universidad Tecnológica ECOTEC formally authorizes the scientific dissemination of the anonymized dataset “ECOTEC_Online Student Risk Dataset,” as well as its use for research, methodological validation, scientific reproducibility, academic training, and the development of evidence-based solutions, in accordance with institutional policies and applicable regulations.

In witness thereof, this certification is duly signed.

Edgar Salas, Ph.D.
Director, ECOTEC Online
Universidad ECOTEC
